# Optimal soak times for Baited Remote Underwater Video Station surveys of reef-associated elasmobranchs

**DOI:** 10.1371/journal.pone.0231688

**Published:** 2020-05-08

**Authors:** Leanne M. Currey-Randall, Mike Cappo, Colin A. Simpfendorfer, Naomi F. Farabaugh, Michelle R. Heupel

**Affiliations:** 1 Australian Institute of Marine Science, Townsville, Queensland, Australia; 2 Centre for Sustainable Tropical Fisheries and Aquaculture & College of Science and Engineering, James Cook University, Townsville, Queensland, Australia; 3 Department of Biological Sciences, Florida International University, Miami, Florida, United States of America; 4 Integrated Marine Observing System (IMOS), University of Tasmania, Hobart, Tasmania, Australia; Swansea University, UNITED KINGDOM

## Abstract

Effective sampling of marine communities is essential to provide robust estimates of species richness and abundance. Baited Remote Underwater Video Stations (BRUVS) are a useful tool in assessment of fish assemblages, but research on the optimal sampling period required to record common and rare elasmobranch species is limited. An appropriate ‘soak time’ (time elapsed between settlement of the BRUVS on the seabed and when it is hauled off the seabed) requires consideration, since longer soak times may be required to record species rare in occurrence, or sightings in areas of generally low elasmobranch abundance. We analysed 5352 BRUVS deployments with a range of soak times across 21 countries in the Coral Triangle and Pacific Ocean, to determine the optimal soak time required for sampling reef-associated elasmobranchs, considering species rarity, and community abundance at each site. Species were categorised into 4 ‘rarity’ groups (very rare to common), by their relative occurrence in the dataset, defined simply by the proportion of BRUVS on which they occurred. Individual BRUVS were categorised into 3 ‘abundance’ groups (low to high) by overall relative elasmobranch abundance, defined as total number of all elasmobranchs sighted per unit of sampling effort. The effects of BRUVS soak times, and levels of rarity and abundance groupings, on the time to first sighting (*TFS*) and time to maximum number of elasmobranchs observed (*t*_*MaxN*_) were examined. We found that *TFS* occurred earlier for species groups with high occurrence, and on BRUVS with high elasmobranch abundance, yet longer soak times were not essential to observe rarer species. Our models indicated an optimum of 95% of both sighting event types (*TFS*, *t*_*MaxN*_) was recorded within 63–77 minutes, and a soak time of 60 minutes recorded 78–94% of the elasmobranch sighting events recorded (78–94% of *TFS* events and 82–90% of *t*_*MaxN*_ events), when species rarity and abundance on BRUVS was accounted for. Our study shows that deployments of ~ 77 minutes are optimal for recording all species we observed, although 60 minutes soak time effectively samples the majority of elasmobranch species in shallow coral reef habitats using BRUVS.

## Introduction

The abundance of elasmobranchs can be highly variable [1, 2] and will be dependent on species distributions, environmental conditions, and local fishing pressure [3, 4]. Their distributions can cross political borders and concerns about conservation status of some species can clash with the interests of commercial and recreational fisheries where they are seen as targets for exploitation [5, 6] or competitors for teleost resources (e.g. [7]). It is therefore important to have a reliable, defensible methodology to make confident comparisons of elasmobranch status within and among locations and jurisdictions.

There are a variety of fishery-independent methods available for estimating the abundance of elasmobranchs [8]. Two of the most common for tropical sharks are underwater visual census (UVC) and baited remote underwater video stations (BRUVS). Both methods have been used in coral reef habitats with UVC most commonly applied to enumerate reef sharks [9–12]. However, BRUVS have been increasingly applied to assessment of shark assemblages over the last two decades [1, 13–16].

One of the main benefits of the baited video technique is that it removes the need for skilled observers in the field, yet is easily standardised for application to a variety of locations and depths. UVC requires a scuba diver highly skilled in fish identification to conduct each survey and there is no visual record of the sample. The presence of video footage permits visualisation of the seascapes in the field of view and species identifications in an archive that can be interrogated repeatedly for a variety of research questions [17]. Due to the use of bait as an attractant, easy replication, and capacity to sample a variety of depths and habitats, BRUVS are an ideal method for studying broad-scale presence and abundance of elasmobranch species without capturing and handling animals (e.g. [2, 14, 18–21]).

The status of reef-associated elasmobranchs has been the subject of numerous studies which have produced varying perspectives about their conservation status and the efficacy of their management [2, 5, 22]. However, the currently available data are heavily biased to carcharhinid sharks with relatively little known about the status of other elasmobranchs that use coral reefs [23]. Reports of declines in the abundance of reef-associated sharks due to overfishing (e.g. [9, 11, 22]) have focussed the use of BRUVS surveys to detect effects of marine protected areas (MPA). Several studies have revealed increased numbers of elasmobranchs in areas closed to fishing [16, 21, 24, 25] indicating the efficacy of BRUVS to detect the effects of fishing. However, there may also be sources of variability in the size of reef shark populations beyond those related to fishing. For example, highly variable baseline estimates of sharks were recorded at a number of Pacific Ocean reefs which were largely attributed to differences in ocean productivity [26]. Further study over broad spatial scales is required to help refine our understanding of variability in reef elasmobranch populations and the factors driving these patterns. This requires capacity to assess relative abundance and compare communities among a variety of sites in a standardised way.

The application of BRUVS provides an opportunity to sample a wide array of depths and habitat types using multiple units simultaneously (e.g. lagoon, slope, inter-reef habitats) and to survey large areas more efficiently than UVC surveys. A large number of BRUVS can be deployed in quick succession to sample a broader area than is surveyed in a UVC transect. The amount of time a BRUVS is deployed on the seabed (‘soak time’) is constrained by the travelling time between sampling sites, the handling time in setting and retrieving the units, camera storage capacity and battery life. A common presumption is that longer soak times would be needed in areas of low abundance (e.g. [27]), and that differing soak times could produce differing results depending on how readily elasmobranchs are attracted to the BRUVS.

Although sharks and rays are easily attracted to bait, it is unclear how they interact with BRUVS units. It could be assumed that in areas of high abundance BRUVS are likely to be placed in proximity to local sharks. In these instances individuals could be expected to appear in the BRUVS field of view early in the deployment, and accumulate to high numbers. In contrast, it could also be assumed that in areas of low overall elasmobranch abundance where individuals are sparsely distributed, or for particular species that are rarely encountered in the region, that likelihood of deploying a BRUVS in their proximity is low and hence arrival times would be longer (e.g. [28]). For example, a soak time of 5–6 hours was suggested as appropriate for detecting the presence of nearshore white sharks *Carcharodon carcharias*, since their mean time to first sighting was 148 minutes for a maximum of one individual at any time [20]. While 1 hour soak times have been suggested for sharks [8], these assumptions have not been tested across multiple locations, and fundamental questions about how BRUVS accumulate sightings of elasmobranchs warrant exploration.

The Global FinPrint project (https://globalfinprint.org/) has conducted BRUVS deployments in coral reef ecosystems around the world to estimate the relative abundance of reef-associated elasmobranchs. Countries within the Coral Triangle and Pacific Ocean comprise a wide diversity and abundance of elasmobranchs, with species varying in rarity (i.e. occurrence on BRUVS), and sites ranging from low to high overall abundance (i.e. overall elasmobranch sightings per unit effort for each BRUVS). We examined this data to identify the optimal soak time required to capture the events of the time to first sighting (*TFS*) and time to maximum number of elasmobranchs observed (*t*_*MaxN*_), compared with the commonly used soak time of 60 minutes. The soak time required to effectively capture these sighting events was assessed under varying levels (categories) of: (1) species rarity: the occurrence of each species on the individual BRUVS in the dataset, grouped into categories of species rarity; and (2) overall elasmobranch abundance on individual BRUVS. These findings provide a basis and recommendations for future research using BRUVS to help define the occurrence and relative abundance of tropical, reef-associated elasmobranchs.

## Materials and methods

**Fig 1:**
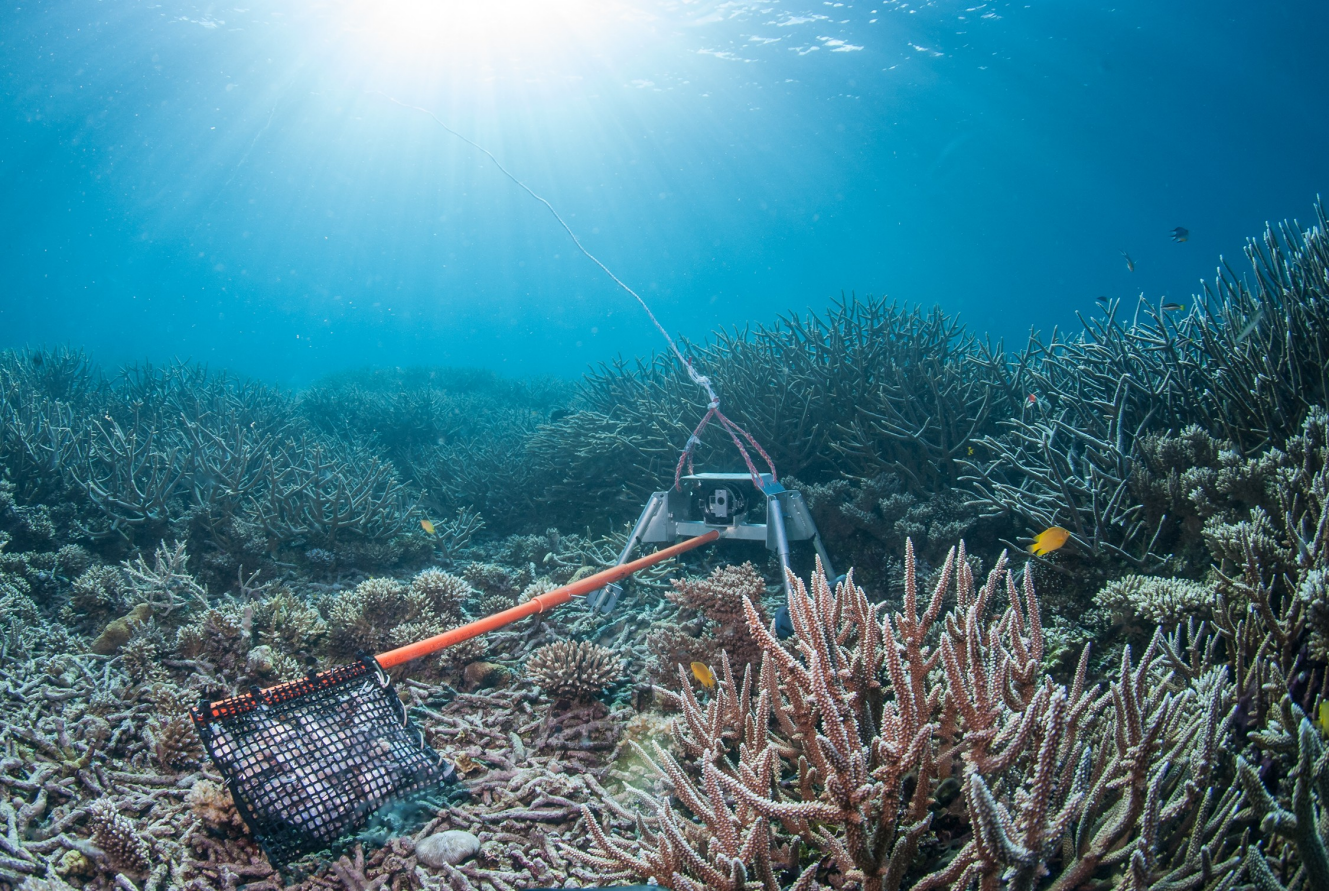
BRUVS deployed on seabed in reef habitat. BRUVS showing bait arm, camera within housing and rope attached to surface float. Credit: S Lindfield, Coral Reef Research Foundation Palau.

Our study included data from 5352 baited remote underwater video station (BRUVS) deployments of the Global FinPrint project in 21 countries. Deployments were conducted in coral reef habitats in depths from 1–70 m. Each BRUVS consisted of a lightweight aluminium frame fitted with a video camera (GoPro Hero4 Silver, GoPro Inc. USA; https://www.gopro.com) within a housing overlooking a bait bag positioned 1.5 m from the camera (Fig 1). Each BRUVS was baited with approximately 1 kg of oily fish (primarily from families Clupeidae and Scombridae). BRUVS were separated by at least 500 m at any time to reduce the likelihood of individuals occurring on multiple cameras [29].

### Video and data analysis

Data comprised standardised legacy data and records from videos analysed using the FinPrint Annotator (v.1.1.44.0) which were examined by two readers, with reader discrepancies, outliers and a subset of images reviewed by a master annotator to ensure accuracy in species identification. Elasmobranchs were identified to species, and the maximum number of individuals of each species seen together in one frame (‘*MaxN*’, a conservative measure of relative abundance [29]), was recorded for each BRUVS video. Videos were analysed from the time the BRUVS settled BRUVS on the seabed (start time), with the ‘time to first sighting’ (*TFS*) recorded for each species, and *MaxN* updated if the number increased at a later point in the video.

Four measurements obtained from BRUVS were used in this study: the ‘soak time’; ‘time to first sighting’ (*TFS*); *MaxN*; and ‘time to *MaxN*’ (*t*_*MaxN*_). The ‘soak time’ was measured as the minutes elapsed between the start time and the instant at which the BRUVS was hauled off the seabed (the end time). For instances where bait was entirely removed from a BRUVS, the time at which this occurred was classified as the end time and marked completion of the soak, for standardisation among BRUVS. Elasmobranch observations beyond the end time were not included in analyses. The ‘time to first sighting’ (*TFS*) was the time elapsed between the start of the sampling period and the first record of a particular species in the field of view. The ‘time to *MaxN*’ (*t*_*MaxN*_) for each species was the time elapsed between the start of the sampling period and the *MaxN* event, representing the time at which maximum relative abundance was observed. These metrics have been reviewed [17, 29] and this study focuses on comparisons between event timing and soak time from the two datasets: *TFS* data; and *t*_*MaxN*_ data, which includes an additional 1337 BRUVS, for which only *MaxN* for each species was recorded (S1 Table). All times were measured in decimal minutes, rounded upward to the nearest whole minute for all analyses.

**Fig 2:**
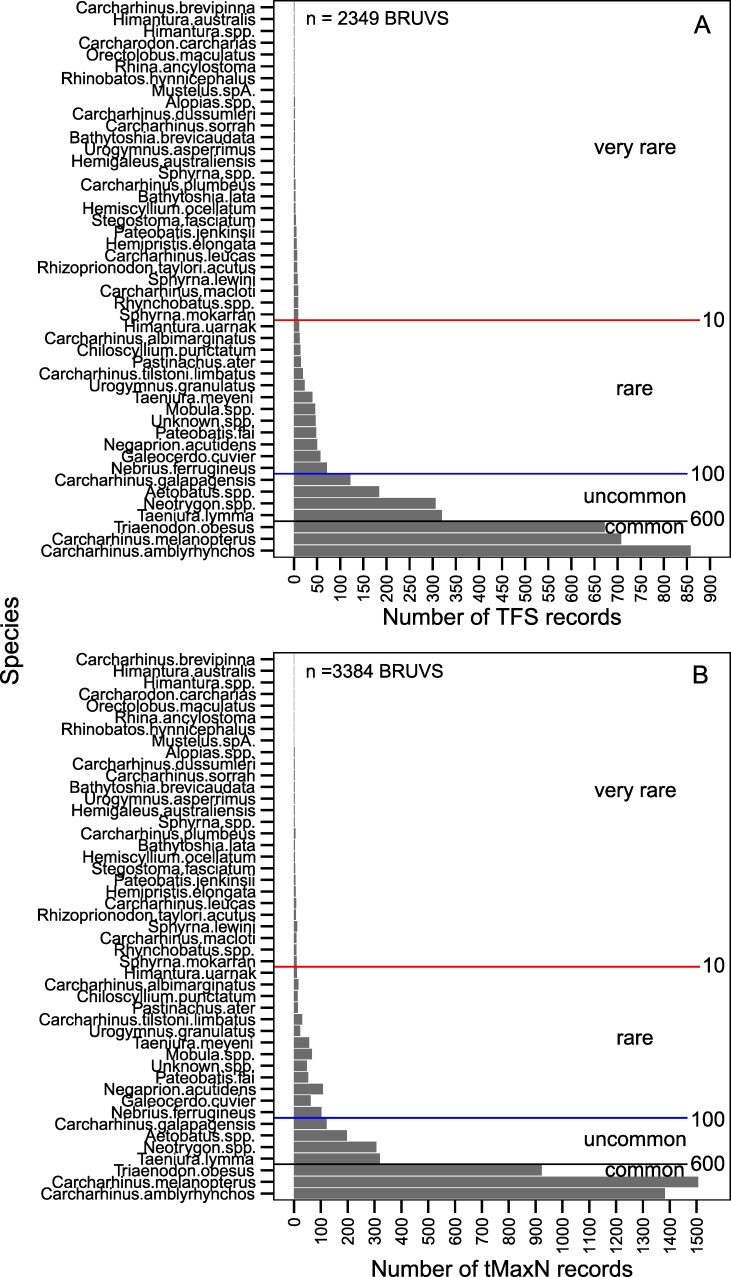
Relative occurrence of 47 species of elasmobranchs in the *TFS* dataset (A; *n* = 2349) and *t*_*MaxN*_ dataset (B; *n* = 3384). Species are ranked from top to bottom by the number of records in the *TFS* dataset. Species were grouped for some analyses by the cut points shown for levels of rarity: ‘very rare’ (occurrence on ≤ 10 BRUVS); ‘rare’ (11–100), ‘uncommon’ (101–600); and ‘common’ (≥ 600 BRUVS).

Relative elasmobranch abundance for each BRUVS sample was calculated as sightings per unit effort (*SPUE*), being *MaxN* values summed across species on individual BRUVS divided by the soak time, multiplied by 60 to provide an hourly rate. *SPUE* was used to assign three categories of overall elasmobranch abundance on individual BRUVS: ‘low’ (*SPUE* ≤ 1); ‘medium’ (*SPUE* > 1 and *SPUE* ≤ 3, which incorporated the global, median *SPUE*) and ‘high’ (*SPUE* > 3). Records for each species were assigned to four levels of ‘occurrence’ (‘rarity’) based on the number of BRUVS samples on which they were recorded in the *TFS* dataset (2349 BRUVS, Fig 2). These species categories were: ‘very rare’ (occurrence on ≤ 10 BRUVS), ‘rare’ (11–100), ‘uncommon’ (101–600), and ‘common’ (> 600). Rarity and abundance groups were used as predictors with soak time in the analyses, and all analyses were conducted in the R environment [30].

#### Summaries of proportions of events by time

Where cumulative frequency shows the frequency of an event in an interval, the empirical cumulative density function (*ECDF*, a step function) was used to estimate the fraction of observations of *TFS* and *t*_*MaxN*_ that were less than or equal to a specified value (using the ecdf function in R). The *ECDF* plots were used to find the 75^th^ and 95^th^ percentiles of *TFS* and *t*_*MaxN*_ by species occurrence (4 levels of rarity), and by abundance on individual BRUVS (3 levels based on *SPUE*).

#### Modelling the effect of soak time, rarity and abundance on timing of events

To identify patterns in *TFS* and *t*_*MaxN*_ with soak time, species rarity, and overall abundance, Aggregated Boosted Regression Trees (ABT, using abt package in R: [31, 32]) were used. ABTs modelled the relationship between the *TFS* and *t*_*MaxN*_ events with the predictors: species rarity (4 levels based on occurrence); overall elasmobranch abundance (3 levels based on *SPUE* per BRUVS); and soak time. We identified the optimal soak times, and considered the commonly used soak time of 60 minutes as sufficient if ABTs indicated the majority of events occurred within 60 minutes, i.e. for all categories of abundance and species rarity. ABTs use the benefits of both regression techniques and classification by machine learning to derive a single, optimal tree by cross-validation (see [33, 34]). The final ABT object is a collection of five trees and results are presented as partial effects plots for the main effects of soak time, abundance and species rarity groups, and partial interaction plots of the effect of soak time by abundance group and soak time by rarity group. The ABT approach is ideal for large datasets with missing values, large outliers, correlated predictors and unequal sample sizes. While regression models summarise how much variation is explained by model fits, ABTs summarise how much variation is predicted, and an equivalent of the pseudo-R-squared value from regression is produced [33].

BRUVS were excluded from the ABT analyses if soak time > 126 (i.e. 2.1% of total BRUVS with > 2 hours and 6 minutes), due to scarcity of cases with very long soaks beyond this time. Responses were transformed by 4^th^ root to reduce the skew in the data, and the models were event^0.25^ ~ soak time + species rarity group + abundance group. Five-fold cross-validation was used for all models, and a range of learning rates from 0.01 (fast) to 0.001 (very slow) were tested with tree sizes of 1000 and 5000.

## Results

There were 47 elasmobranch species recorded from 21 countries in 5352 BRUVS samples, with sightings on 63.2% of the deployments (S1 Table). Information on *TFS* was available for 2349 BRUVS and data were obtained for *t*_*MaxN*_ from 3384 BRUVS (S1 Table).

The *MaxN* recorded for each species was highly skewed, with an overall median of *MaxN* = 1, a maximum of 21, and a mean of 1.6. The very high values were caused by passage of schools of rays or sharks through the field of view. Total relative abundance per BRUVS ranged from 1 to 32, with a median of 2 and a mean of 2.5 for *t*_*MaxN*_ data. The predominance of *MaxN* = 1 implied that *t*_*MaxN*_ was equivalent to *TFS* for most species and most BRUVS. The overall *SPUE* per BRUVS ranged from 0.2 to 62.4, with a median of 1.5 sighting hr^-1^ and mean of 2.4 sighting hr^-1^. Soak times for seven of the eight BRUVS with SPUE >25 were of 30 minutes soak time or less, caused by removal of the bait by predators.

### Timing of events based on species rarity

Three species comprised the ‘common’ category of species rarity based on occurrence: grey reef shark (*Carcharhinus amblyrhynchos*), blacktip reef shark (*C*. *melanopterus*), and whitetip reef shark (*Triaenodon obesus*) (Fig 2). The ‘uncommon’ category included three rays and one shark species: spotted fantail ray (*Taenura lymma*), maskrays (*Neotrygon* spp.), eagle rays (*Aetobatus* spp.), and the Galapagos shark (*Carcharhinus galapagensis*) (Fig 2). Most other species were classified as ‘very rare’ (27) or ‘rare’ (13) and occurred on 86 (3.7%) and 418 (17.8%) of the 2349 BRUVS in the *TFS* dataset, respectively. The most common species, *C*. *amblyrhynchos*, was recorded on 858 (36.5%) of the 2349 BRUVS where *TFS* information was available, 1382 of the 3384 BRUVS with *T*_*MaxN*_ data (40.8%; Fig 2A and 2B), and had an overall recorded abundance (total summed *MaxN*) of 2989 individuals. The larger *t*_*MaxN*_ dataset (Fig 2B) showed similar rankings due to the prevalence of *MaxN* = 1 for most species and most BRUVS (e.g. S1 Fig).

**Fig 3:**
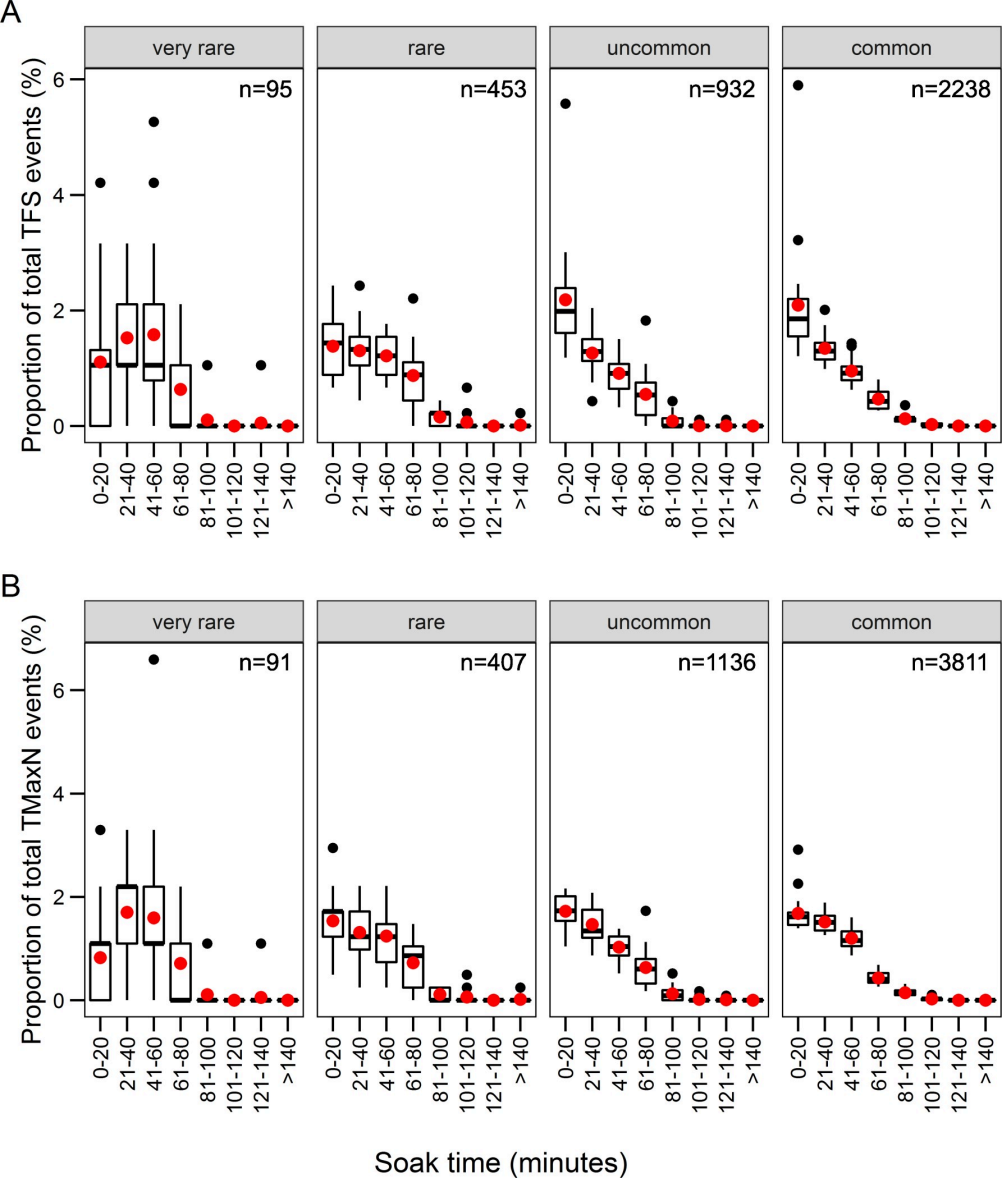
Proportions of species in rarity groups by soak time (minutes). Species groups were categorised as ‘very rare’ (occurrence on ≤ 10 BRUVS), ‘rare’ (11–100), ‘uncommon’ (101–600), and ‘common’ (≥ 600 BRUVS) species in the *TFS* dataset (A; *n* = 2349) and *t*_*MaxN*_ (B; *n* = 3384) dataset (see Fig 2A). The boxes show the medians (black lines) and means (red points) within the first and third quartiles of the data. The ‘whiskers’ represent 1.5 times the interquartile ranges, and outliers in events proportions are shown.

**Fig 4:**
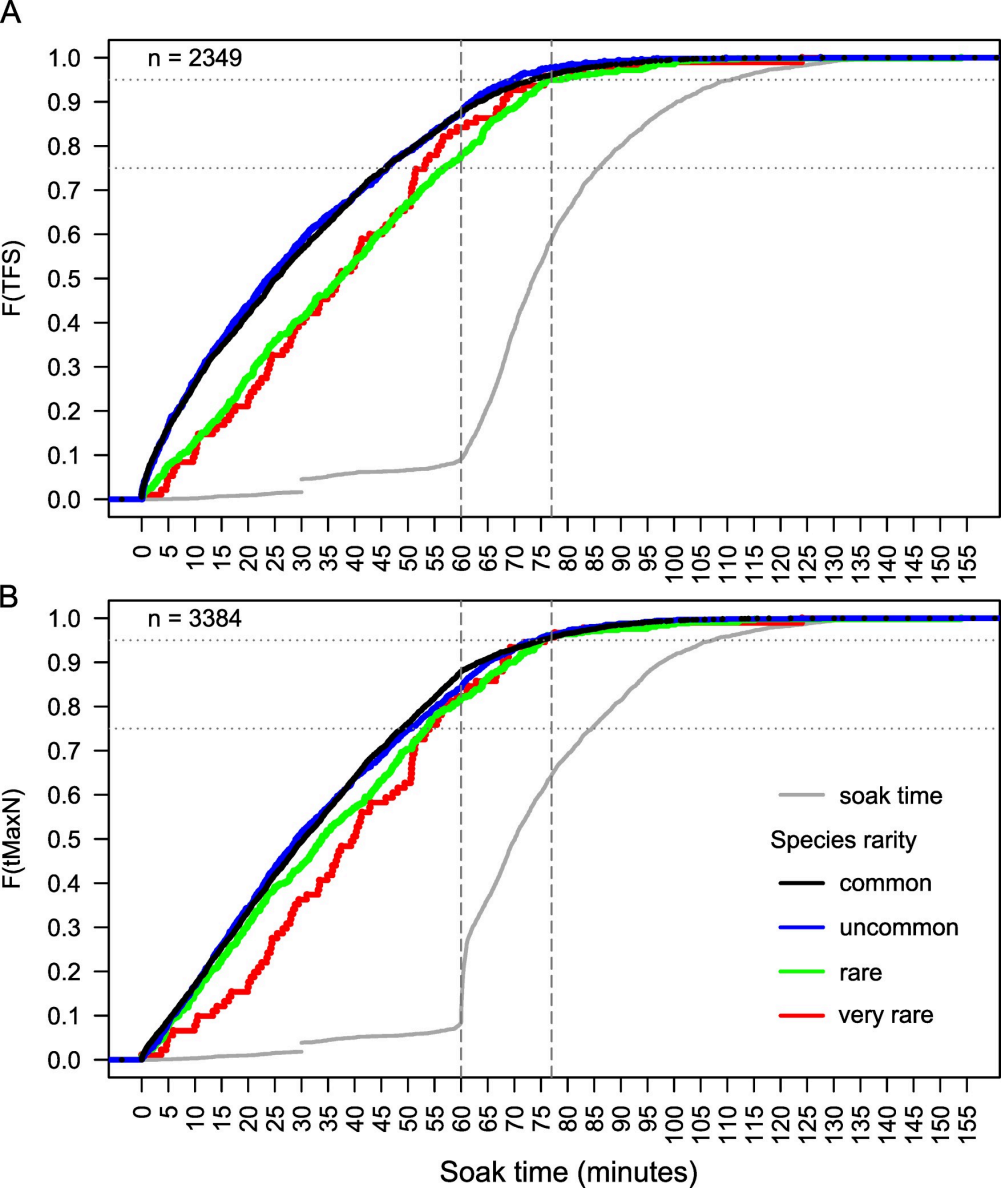
Time to first sighting (*TFS*: A) and time to maximum *MaxN* (*t*_*MaxN*_: B) by species rarity group. Empirical cumulative density functions (*ECDF*) of the *TFS* and *t*_*MaxN*_ by 4 species groups based on their rarity (see Fig 2A). The *ECDF* for soak times are also shown for each dataset. Horizontal lines on the y-axis show 75^th^ and 95^th^ percentiles in the *ECDF*. Vertical lines on the x-axis represents a soak time of 60 and 77 minutes.

For each level of species rarity, counts of *TFS* and *T*_*MaxN*_ event observations for each minute were calculated and represented as a proportion of the total observations per level of rarity. Boxplots of the event proportions observed per minute for 20 minute time bins showed no evidence that rarer species were being sighted later in videos, or that the highest proportions peaked later than those of the more common categories (Fig 3). While the proportion of events was lower in the first 0–20 minutes for the ‘very rare’ species compared to other three rarity groups, the greatest proportion of all species groups based on rarity were observed within the first 60 minutes of soak time, with < 60 minutes comprising the majority of *TFS* and *T*_*MaxN*_ events. However, empirical cumulative density functions for the four categories of species rarity gave a clearer picture separating the two most common species groups from the other two groups (Fig 4). About 78–88% of all *TFS* events and 82–88% of all *t*_*MaxN*_ events occurred for all species groups by 60 minutes soak time, and 95% of recorded *TFS* and *t*_*MaxN*_ events for all species groups had occurred by ~ 77 minutes. ‘Common’ and ‘uncommon’ species had almost identical cumulative density functions for both *TFS* and *t*_*MaxN*_, which occurred earlier than for ‘rare’ and ‘very rare’ species. The timing of *t*_*MaxN*_ was delayed by about 10 minutes for ‘very rare’ species between about 10 and 50 minutes soak time, after which the curves converged (Fig 4B).

### Timing of events based on relative abundance

**Fig 5:**
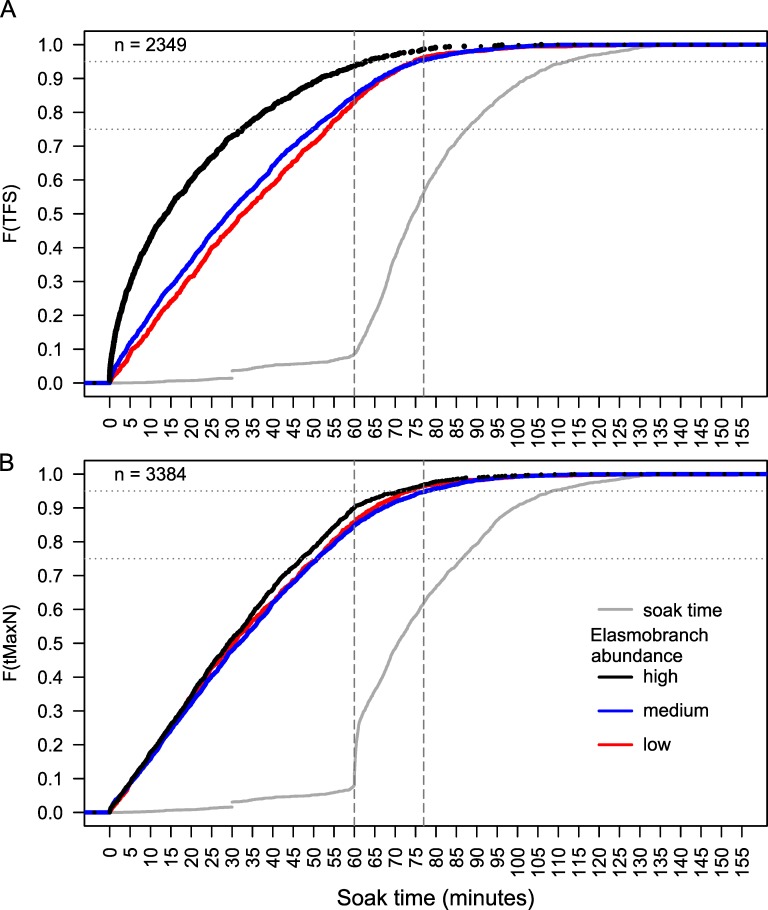
Time to first sighting (*TFS*: A) and time to maximum *MaxN* (*t*_*MaxN*_: B) by abundance group. Empirical cumulative density functions (*ECDF*) of the *TFS* and *t*_*MaxN*_ by 3 groups based on relative abundance of all elasmobranchs (*SPUE*) per BRUVS. The *ECDF* for soak times are also shown for each dataset. Horizontal lines on the y-axis show 75^th^ and 95^th^ percentiles in the *ECDF*. Vertical lines on the x-axis represents a soak time of 60 and 77 minutes.

Approximately 83–94% of all *TFS* events and 85–90% of all *t*_*MaxN*_ events occurred by 60 minutes of soak time for BRUVS of all elasmobranch abundance (*SPUE*) groups (Fig 5). At least 95% of all *TFS* and *t*_*MaxN*_ events for all groups had occurred by ~ 77 minutes soak time. Elasmobranchs were sighted faster (10–15 minutes earlier) on BRUVS where higher overall abundance was recorded, compared to BRUVS with medium and low *SPUE*, particularly within the first 60 minutes (Fig 5A). Thus, a shorter optimal soak time of 63 minutes captured 95% of *TFS* events from BRUVS categorised by high elasmobranch abundance (Fig 5A), linked also to a number of sightings recorded immediately on BRUVS settlement to the seafloor. Little difference among abundance groups was observed for *t*_*MaxN*_ events, with marginal difference between the high versus medium and low curves (Fig 5B).

### Correlations between soak time and events

**Fig 6:**
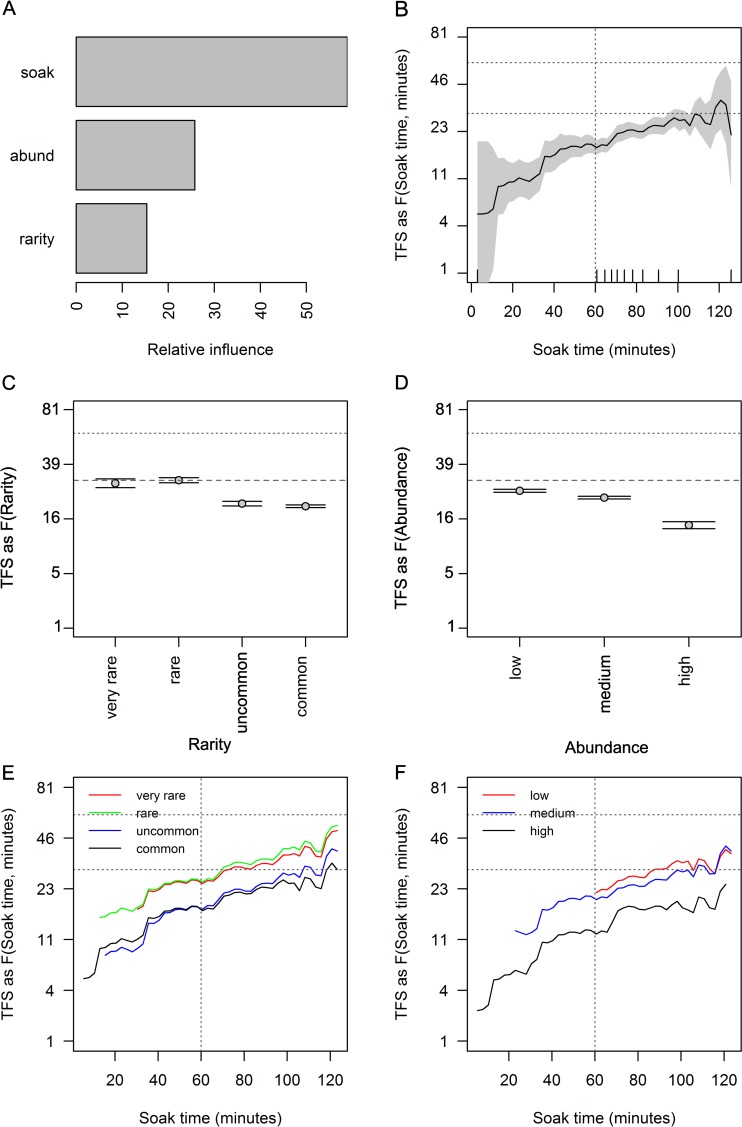
Partial effects plots of soak time, species rarity and abundance groups on *TFS*. Relative influence of each predictor (A), the effects of each predictor: soak time (B); species rarity groups (based on occurrence, C); and abundance groups (based on *SPUE* per BRUVS, D); are shown when the other predictors were held to their average value. The partial effect of soak time given the 4 species rarity groups (E) and 3 abundance groups (F) are indicated. Shading and errors bars are 2 standard errors. The y axes are back-transformed responses from the 4^th^ root scale, expressed in minutes. The rugs on the x-axis of panel (B) are ten percentiles in the distribution of the soak times. Horizontal lines on panels show mean *TFS* (30.6 minutes) and 60 minutes and vertical lines indicate a soak time of 60 minutes.

**Fig 7:**
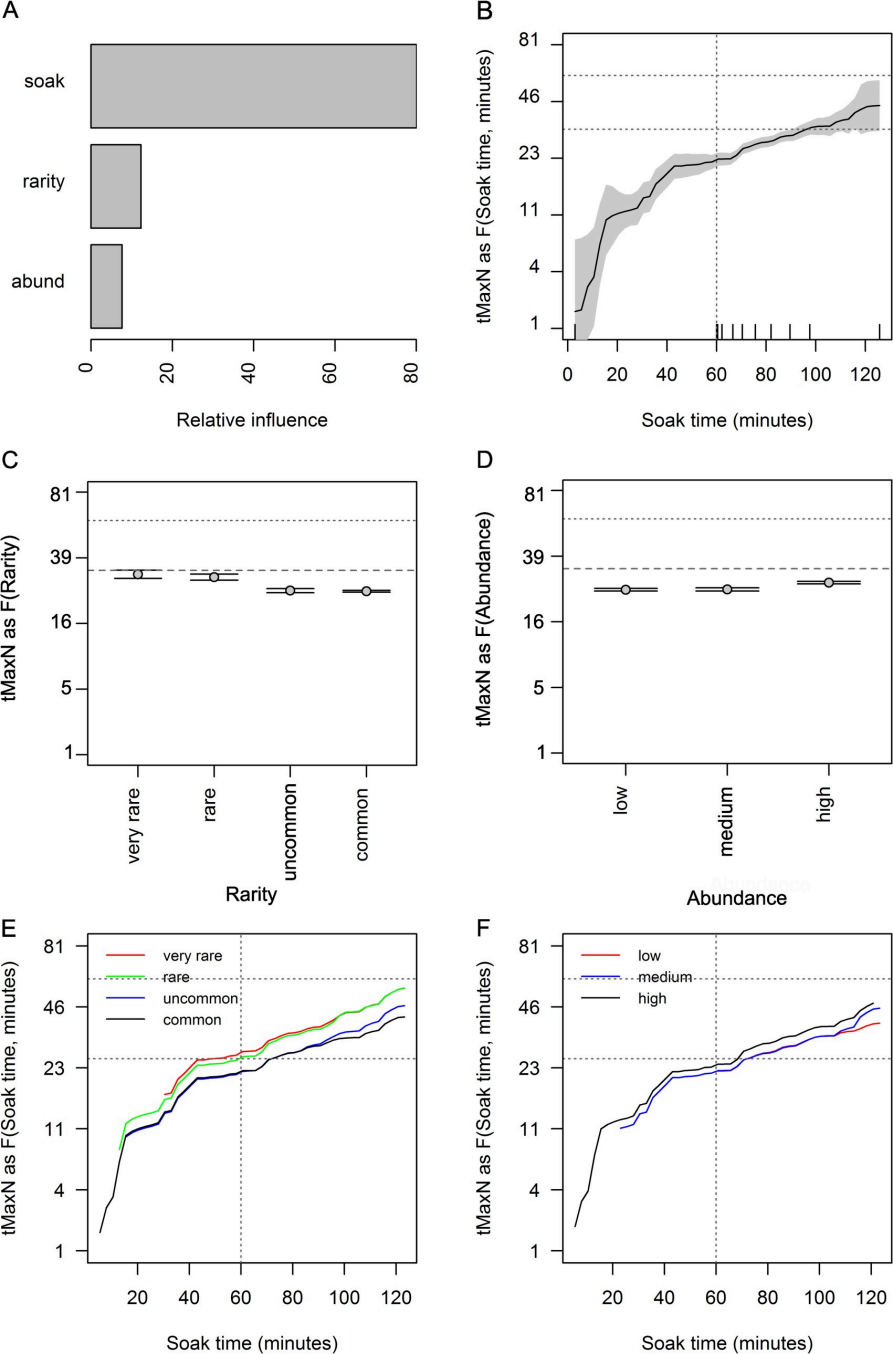
Partial effects plots of soak time, species rarity and abundance groups on *t*_*MaxN*_. Relative influence of each predictor (A), the effects of each predictor: soak time (B); species rarity groups (based on occurrence, C); and abundance groups (based on *SPUE* per BRUVS, D); are shown when the other predictors were held to their average value. The partial effect of soak time given the 4 species rarity groups (E) and 3 abundance groups (F) are indicated. Horizontal lines show mean *t*_*MaxN*_ (33.5 minutes) and 60 minutes. All other conventions follow Fig 6.

The transformed events of *TFS* (*n* = 3652 on 2314 BRUVS) and *t*_*MaxN*_ (*n* = 5393 on 3347 BRUVS) for 47 species were modelled as responses to the predictors soak time, species rarity and abundance groups for ABT analyses. Less than ~ 10% of soak times ended before ~ 60 minutes, and less than 10% of soaks were longer than ~ 100 minutes (Figs 6B and 7B). Mean soak times in the datasets (< 126 minutes) for *TFS* and *t*_*MaxN*_ were 76 and 73.5 minutes respectively. The global, mean *TFS* and *t*_*MaxN*_ occurred at less than half of these values at 30.6 and 33.5 minutes, indicating mean values had been reached within the first half of most deployments. There were positive correlations of 0.30 and 0.27 between soak time and *TFS* and soak time and *t*_*MaxN*_.

### The effect of soak time on time to first sighting *(TFS)*

The best model for *TFS* incorporated all three predictors with second-order interactions (depth = 3). The relative prediction error of 0.85, equated to an R-squared of only 15.3%, and the relative influence of soak was 58.9%, with a generally increasing relationship between soak time and mean predicted *TFS* (species and BRUVS pooled) (Fig 6A and 6B). The relative influence of abundance group was 25.8% and 15.3% for rarity. The slope in the response of *TFS*^0.25^ to soak time was generally positive, but relatively small over the range of soak times where 80% of the data occurred, i.e. ~ 9 minutes between 60 and 100 minutes (Fig 6B).

For BRUVS where elasmobranchs were classified as high in relative abundance, the *TFS* for a given soak time was observed faster than for BRUVS where *SPUE* of elasmobranchs were low to medium (Fig 6D and 6F). Likewise, predicted *TFS* for the common and uncommon rarity groups were earlier than for the rare and very rare groups (Fig 6C and 6E). The curves for each group ended below *TFS* = 60 minutes and majority below ~ 46 minutes, for the entire set of soak times up to 126 minutes (Fig 6F), although the uncertainty was high in the top 10 percent of soak times (Fig 6B).

### The effect of soak time on time to *MaxN* (*t*_*MaxN*_)

The best model for *t*_*MaxN*_ incorporated all three predictors (soak time, rarity and abundance groups with interaction depth = 2), with a high relative prediction error (0.94), equating to a very small R-squared of 6.3%. The model for *t*_*MaxN*_ with soak time alone had a marginally lower R-squared of 6.1%. The relative influence soak time was 80.1%, with relative influence of 12.3% and 7.6% for rarity and abundance groups respectively (Fig 7A). There was a positive increase in *t*_*MaxN*_ of about 8 minutes between soak times of 60 and 90 minutes, with greater uncertainty and fewer samples for soak times beyond 100 minutes (Fig 7B). Little difference in *t*_*MaxN*_ was observed by rarity or abundance groups (Fig 7C–7F). Although soak time explained a small proportion of the overall model deviance, this analysis indicates *t*_*MaxN*_ events were observed within 60 minutes of soak time, and the majority of the curves predicted *t*_*MaxN*_ events occurred prior to a *t*_*MaxN*_ ~ 46 minutes (Fig 7B, 7E and 7F).

## Discussion

Optimal soak times for BRUVS are a trade-off between logistical constraints and statistical performance to detect differences in selected metrics between samples. Logistical challenges include balancing handling time of the hardware in the field, the need to maximise spatial replication whilst maintaining independence of samples, and the desire to minimise video interrogation times in the laboratory. Statistical constraints include the desire to reach asymptotes in species accumulation curves or species counts [35, 36], minimise the ratio of the standard error to the mean of the metrics of interest (‘precision’, *sensu* [37]), maximise the ‘power’ to detect differences amongst treatments [38, 39, 40], or minimise mis-classification rates of models on a validation data set [41]. All four of these statistical constraints have been examined for fish assemblages recorded by different BRUVS soak times (see [27, 42–46]). Yet, for elasmobranchs in coral reef environments, an optimal soak time has been lacking. Based on our results, first sightings occurred earlier for species groups with high occurrence, and on BRUVS with high elasmobranch *SPUE*, yet sightings of rarer species were not restricted to longer soak times. A soak time of 60–77 minutes recorded 78%-95% of all sighting records for reef elasmobranchs regardless of species rarity, and BRUVS-level abundance. These results confirm that a BRUVS soak time of ~ 77 minutes is optimal, while 60 minutes sufficiently captures the majority of sightings relevant for reef-associated elasmobranch community surveys.

Our ability to test the effect of varying soak times on these metrics for elasmobranchs extends research on the dynamics of visits to baited cameras by teleosts. Soak times for teleost community studies typically range from 30 to 90 minutes [47] and are most commonly 60 minutes by Australian standard [48, 49]. Thus most evaluations of optimal soak times have been confined to these limits, but results vary by habitat type. For example, A soak time of 15 minutes was the shortest duration able to capture abundance and length metrics for Hawaiian ‘bottom fish’, whilst 30 and 40 minute soak times generated data that did not significantly differ [45]. Another comparison of soak times (20, 40 and 60 minutes) in terms of species richness, *MaxN*, times to first arrival and times to *MaxN* values for Hawaiian bottom fish assemblages, found soak times of 60 minutes were considerably more powerful for detecting statistical differences in sessile ‘macropiscivores’ and ‘generalist macropiscivores’ [42]. Yet either 30 or 60 minute soak times were found to provide a reasonable estimate of rocky reef fish diversity (< 25 species) and relative abundance for comparative purposes [44]. Mean species richness, mean total *MaxN* and mean species-specific *MaxN* did not differ among soak times of 30, 60 and 90 minutes in estuarine habitats, but ‘precision’ rose with increasing sampling time for small fish on seagrass and sandy substrata [43]. Precision of species richness and abundance of a semi-pelagic fish assemblage improved when soak time was extended from 60 to 120 minutes on pelagic stereo-BRUVS, but not when soak times were increased to 180 minutes [27]. Therefore testing of effects of soak time in different habitats and for different species groups is crucial to interpretation of relative abundance data.

Studies using BRUVS to specifically identify and count elasmobranchs have used soak times of 60 minutes [14], 90 minutes [1, 13], 300 minutes [20], and up to 615 minutes [28]. Some of these studies offer inference about the timing of arrival of ‘rare’ species. For example, the time to first appearance of juvenile white sharks (*Carcharodon carcharias*) ranged from 15 to 299 minutes with a mean of 148 ± 15 minutes [20], and mean arrival times of the Greenland shark (*Somniosus microcephalus*) was found between 118 to 280 minutes [28]. These studies focus on species occurring in low numbers, which may require longer soak times. Our tests of the effects of soak time for reef-associated elasmobranchs with boosted regression trees indicated that soak time was not a major driver of events concerning times of arrival and attainment of *MaxN*, accounting for only 9% and 8% of the total predicted variation in *TFS* and *t*_*MaxN*_, respectively. This result likely reflects the high potential for sighting reef-associated elasmobranchs on BRUVS deployed in clear water reef habitats. There were obviously other major factors not analysed here that governed elasmobranch dynamics, such as micro-habitat type in the field of view, reef type and fishing pressure. These factors are important in other studies of reef-associated elasmobranch abundance [1, 16].

Our analyses showed a slight trend for delay in events for a given soak time at sites where overall elasmobranch abundance was classified as ‘low’ or ‘medium’. The times *MaxN* was observed differed little between abundance groups, but for sites characterised by high abundance (*SPUE* > 3 elasmobranchs), *TFS* occurred earlier. This was obvious within the first 60 minutes (94% of events observed), particularly for deployments where elasmobranchs were viewed immediately on settlement of the BRUVS on the seabed. The trend in *TFS* is in accordance with a presumption that arrival times to BRUVS will be faster at sites inhabited by more elasmobranchs. For example, mean arrival times of *S*. *microcephalus* was found to occur within the first half of long (up to 10 hour) soak times with a significant negative exponential relationship between first arrival times and total individuals sighted [28]. In contrast, a four-fold increase in relative abundance of the grey reef shark (*Carcharhinus amblyrhynchos*) on BRUVS was not accompanied by any significant change in *TFS* or *t*_*MaxN*_ in the field of view [16]. Our comparison of soak times across a broad geographic range and groupings based on abundance groups, adds valuable information in this space.

Events (*TFS* and *t*_*MaxN*_) in the Global FinPrint datasets occurred, on average, within ~ 46 minutes in ABT models for all abundance groups, and the mean, global *TFS* and *t*_*MaxN*_ occurred at 30.6 and 33.5 minutes across the variety of soak times recorded. The *ECDF*s indicated that approximately 83–94% of all *TFS* and 85–90% of all *t*_*MaxN*_ events occurred by 60 minutes, and 95% by 77 minutes of soak time, for all abundance groups. Abundance groups also indicated that while first sightings of species occurred faster on BRUVS where elasmobranch *SPUE* was highest, ABT models predicted that all observations were observed within the first 60 minutes. These analyses imply that regardless of BRUVS-specific relative abundance, soak times of 60–77 minutes effectively survey elasmobranch assemblages of associated with shallow, coral reefs in the Pacific and Coral Triangle in daylight hours. These results may also apply to other coral reef systems, as the rates of elasmobranch sightings (*SPUE*) in our abundance groups are comparable to other studies, including the Tubbataha no-take Marine Reserve in the Philippines which was identified as a “hotspot” for elasmobranchs [50].

We found no evidence that rarer species were sighted only on the longest video records or that counts of their maximum number peaked later than those of more abundant species. Species groups classified as ‘very rare’ showed a delay of only ~10 minutes in the timing of *t*_*MaxN*_, but 78–88% of all *TFS* and 82–88% of all *t*_*MaxN*_ events had occurred by 60 minutes, and 95% by 77 minutes soak time, for all species groups. This trend supports an expectation of slightly later arrival of rarer species in the field of view, as BRUVS deployments are more likely to coincidentally overlap with the presence of a common species than a rare one. A soak time of 60 minutes appeared effective in recording the majority of species and individuals, and ~ 77 minutes would be optimal to survey patterns of elasmobranch diversity for all species groups, regardless of rarity. The short delay in arrival of rarest species matches our understanding of the use of habitat by reef-associated elasmobranchs. Tropical ichthyofaunas, are composed of a core group of common species closely associated with coral reef habitat types, and a larger pool of rare, transient species that move through the reefal habitat or use it facultatively (see [51]). Most research has indicated the more common reef sharks (e.g. *C*. *amblyrhynchos*, *C*. *melanopterus*, *T*. *obesus*) rarely move over large distances and tend to have fairly consistent activity spaces on the scale of several kilometres [24, 52–55], and would be obvious members of the core, reef-associated elasmobranch fauna. In contrast, rarer species in our dataset (e.g. tiger sharks, *Galeocerdo cuvier*) may have species-specific habitat preferences, broader movements, or nocturnal foraging patterns that reduce their incidental interaction with the BRUVS field of view (e.g. [56, 57]). The single records of the spinner shark (*C*. *brevipinna*), white shark (*Carcharodon carcharias*), thresher shark (*Alopias* spp.) and the reticulate whipray (*Himantura australis*), amongst many other singletons, could be considered transients. While a ~ 77 minute soak time may be more optimal than 60 minutes for research solely targeting one of these rarer transients in this region, increased replication of 60 minute BRUVS or complementary sampling via other approaches such as 360 degree cameras [58, 59] would likely be beneficial.

While species richness varied among BRUVS, the majority of *MaxN* values were only 1 individual for most species on most BRUVS. This may be because separate visits of single, different, elasmobranchs in the field of view are not recognised in the conservatism of the *MaxN* metric [60]. Low *MaxN* suggests that reef-associated elasmobranchs are not typically found in large groups and/or very high local abundances. Most reef elasmobranchs are not known to aggregate as readily as some pelagic or inshore species (e.g. [61, 62]). Local abundances might also reflect fishing pressure, and most of the sites sampled here have at least some level of fishing pressure, and populations are thought to be in decline in some where targeted fishing of elasmobranchs occurs [26, 63, 64]. The corollary of *MaxN* = 1 is *TFS = t*_*MaxN*_, which limited the contrasts and ranges available in our separate analysis of these metrics. It also prevented species-specific modelling of accumulation of *MaxN* with soak time, which is known to be an indicator of relative abundance when combined with *TFS* [65]. We used *MaxN* in levels of *SPUE* for each BRUVS, and species rarity groupings, as predictors to explain the effects of soak time on the timing of events, and regardless of the reasons behind differences in relative abundance, 95% of *TFS* and *t*_*MaxN*_ events were observed within 63–77 minutes soak time.

## Conclusions and recommendations

We have concluded that a sampling time of 63–77 minutes is optimal for sighting individuals for defining relative abundance of rare to common species, at sites with varying levels of elasmobranch abundance, on shallow tropical reefs across a broad geographic area. Extending deployments and video analysis beyond 60 minutes could have undesirable logistical consequences and questionable benefits, and may not be necessary since 60 minutes appears sufficient to capture the majority of sighting events. Understanding the effectiveness of the commonly used 60 minute soak time is important for robust comparisons among tropical locations, especially for rare or uncommon species. We recommend that researchers routinely record all events relating to timing of first arrival and subsequent timing of increase in *MaxN* to gain the most insight into patterns of relative abundance and the dynamics of elasmobranchs (and teleosts) in the field of view of BRUVS. Unless specifically investigating a single very rare transient species in areas with few elasmobranchs, greater benefit is likely gained through increased replication of 60 minute BRUVS than extending soak times. These metrics are fundamental to addressing questions about the status of populations when using the BRUVS technique, and we provide a baseline for the areas within the regions sampled. We recommend pilot studies compare a range of sampling durations using these methods to gain an understanding of completeness of the biodiversity inventories assessed.

## Supporting information

S1 Table Summary of time to first sighting (*TFS*) and time to *MaxN* (*t*_*MaxN*_) datasets by country in descending order of countries with *t*_*MaxN*_ data. Percentage of BRUVS where elasmobranchs were recorded is shown.
(PDF)

S1 Fig Frequency distribution of *MaxN* for the three most common elasmobranch species observed on BRUVS from the coral triangle and pacific ocean. (PNG)
