# Supplementary material for: Optimal soak times for Baited Remote Underwater Video Station surveys of reef-associated elasmobranchs
Source: PLoS One. 2020 May 8;15(5):e0231688. doi: 10.1371/journal.pone.0231688 (PMC7209308; doi:10.1371/journal.pone.0231688)
Supplement: S1 Table — Percentage of BRUVS where elasmobranchs were recorded is shown. (PDF) [file pone.0231688.s001.pdf]

**S1 Table. Summary of time to first sighting (*TFS*) and time to *MaxN* ($t_{MaxN}$) datasets by country in descending order of countries with $t_{MaxN}$ data. Percentage of BRUVS where elasmobranchs were recorded is shown.**

| Country | Total number of BRUVS | Samples with *TFS* data | Samples with $t_{MaxN}$ data | BRUVS with elasmobranchs |
|---|---|---|---|---|
| French Polynesia | 1201 | - | 962 | 80.1% |
| Australia | 676 | 556 | 556 | 82.2% |
| Indonesia | 806 | 404 | 404 | 50.1% |
| Palau | 216 | 190 | 190 | 88.0% |
| Malaysia | 485 | 185 | 185 | 38.1% |
| Papua New Guinea | 301 | 176 | 176 | 58.5% |
| Federated States of Micronesia | 194 | 172 | 172 | 88.7% |
| USA-Pacific | 125 | 101 | 101 | 80.8% |
| Kiribati | 112 | 84 | 84 | 75.0% |
| Vanuatu | 210 | 79 | 79 | 37.6% |
| Solomon Islands | 106 | 80 | 80 | 75.5% |
| Niue | 98 | 71 | 71 | 72.4% |
| New Caledonia | 74 | - | 62 | 83.8% |
| Fiji | 148 | 53 | 53 | 35.8% |
| American Samoa | 108 | 52 | 52 | 48.1% |
| Samoa | 97 | 42 | 42 | 43.3% |
| Taiwan | 186 | 35 | 35 | 18.8% |
| Japan | 106 | 32 | 32 | 30.2% |
| Tonga | 24 | 22 | 22 | 91.7% |
| New Zealand | 17 | 15 | 15 | 88.2% |
| Cook Islands | 62 | - | 11 | 17.7% |
| TOTALS | 5352 | 2349 | 3384 | 63.2% |
