# Supplementary figures and images for: Optimal soak times for Baited Remote Underwater Video Station surveys of reef-associated elasmobranchs
Source: PLoS One. 2020 May 8;15(5):e0231688. doi: 10.1371/journal.pone.0231688 (PMC7209308; doi:10.1371/journal.pone.0231688)

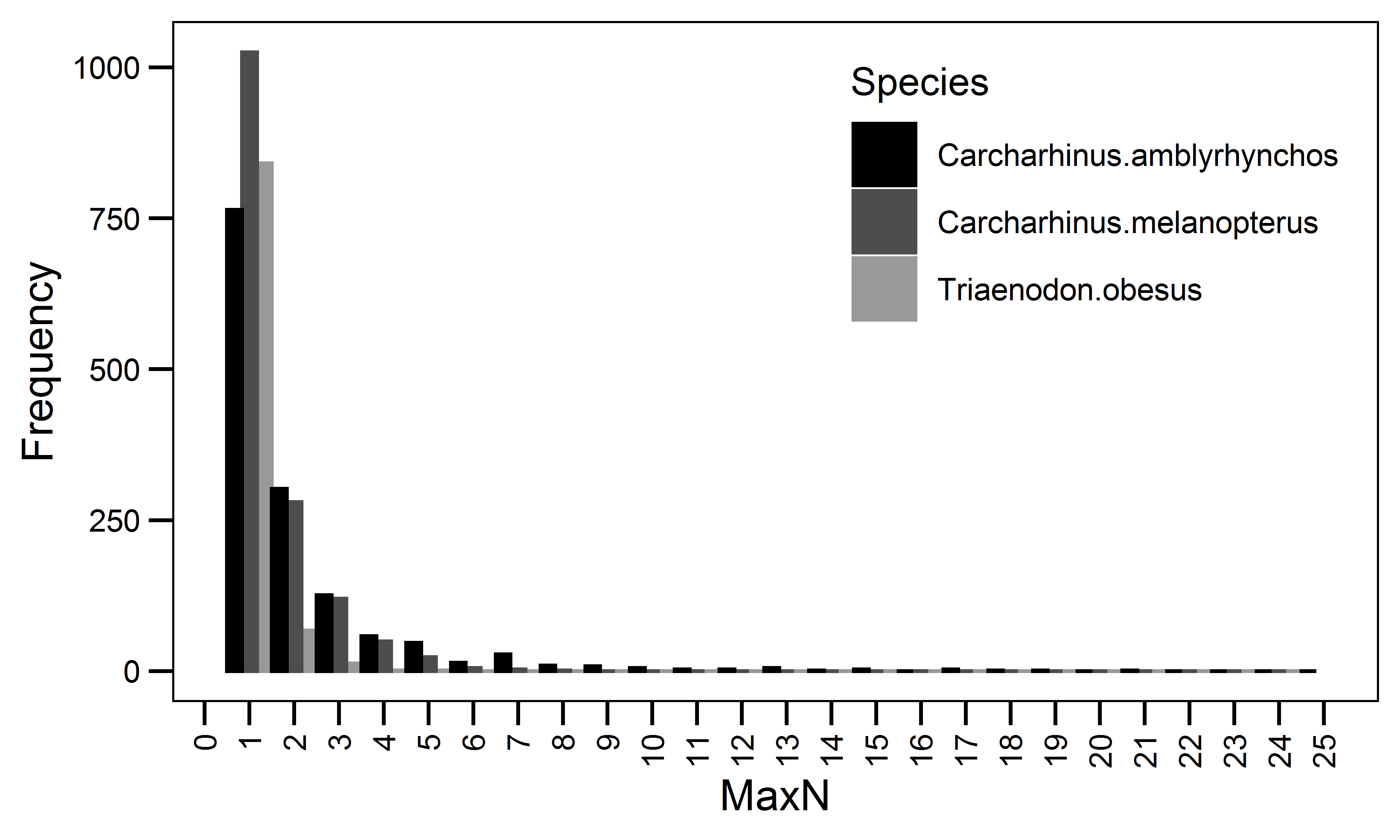

Supplement: S1 Fig — (PNG) [file pone.0231688.s002.png]
